# Damage Proxy Map of the Beirut Explosion on 4th of August 2020 as Observed from the Copernicus Sensors

**DOI:** 10.3390/s20216382

**Published:** 2020-11-09

**Authors:** Athos Agapiou

**Affiliations:** 1 Department of Civil Engineering and Geomatics, Faculty of Engineering and Technology, Cyprus University of Technology, Saripolou 2-8, Limassol 3036, Cyprus; athos.agapiou@cut.ac.cy; 2 Eratosthenes Centre of Excellence, Saripolou 2-8, Limassol 3036, Cyprus

**Keywords:** Copernicus, Sentinel-1, Sentinel-2, InSAR, change detection, damage proxy map, Beirut, Lebanon, explosion

## Abstract

On the 4th of August 2020, a massive explosion occurred in the harbor area of Beirut, Lebanon, killing more than 100 people and damaging numerous buildings in its proximity. The current article aims to showcase how open access and freely distributed satellite data, such as those of the Copernicus radar and optical sensors, can deliver a damage proxy map of this devastating event. Sentinel-1 radar images acquired just prior (the 24th of July 2020) and after the event (5th of August 2020) were processed and analyzed, indicating areas with significant changes of the VV (vertical transmit, vertical receive) and VH (vertical transmit, horizontal receive) backscattering signal. In addition, an Interferometric Synthetic Aperture Radar (InSAR) analysis was performed for both descending (31st of July 2020 and 6th of August 2020) and ascending (29th of July 2020 and 10th of August 2020) orbits of Sentinel-1 images, indicating relative small ground displacements in the area near the harbor. Moreover, low coherence for these images is mapped around the blast zone. The current study uses the Hybrid Pluggable Processing Pipeline (HyP3) cloud-based system provided by the Alaska Satellite Facility (ASF) for the processing of the radar datasets. In addition, medium-resolution Sentinel-2 optical data were used to support thorough visual inspection and Principal Component Analysis (PCA) the damage in the area. While the overall findings are well aligned with other official reports found on the World Wide Web, which were mainly delivered by international space agencies, those reports were generated after the processing of either optical or radar datasets. In contrast, the current communication showcases how both optical and radar satellite data can be parallel used to map other devastating events. The use of open access and freely distributed Sentinel mission data was found very promising for delivering damage proxies maps after devastating events worldwide.

## 1. Introduction

In emerging situations such as industrial and technological accidences, earth observation sensors can provide near-real-time information to local stakeholders and international organizations. This has been already demonstrated in the past in several examples such as the case study of the blast at Cyprus Naval Base (11th of July 2011, at Mari area, Cyprus), at the Fukushima Daiichi nuclear disaster (11th of March 2011, Ōkuma, Japan), etc. High-resolution satellite sensors are mainly used for rapid mapping and recording of the damage over large extents. However, the use of freely distributed sensors such as those of the Copernicus Programme did not thoroughly investigate in the past. This is in contrast with their wide use in monitoring other hazards all over the world, which has been demonstrated in several articles [1,2,3,4]. This communication article aims to investigate whether the use of these sensors, namely the Sentinel-1 and Sentinel-2, can provide reliable information, in case of emerging situations. For this reason, a recent catastrophic event was examined in Lebanon, aiming to produce Damage Proxy Maps (DPM) over the area.

The event took place in the evening of the 4th of August 2020 in the area near the main harbor of Beirut, the capital of Lebanon. According to the media, a fire near the port ignited a large nearby store of ammonium nitrate, which is a highly explosive chemical often used in fertilizer. More than 100 people died, while more than 5000 were wounded. It was estimated that at least 300,000 people were left homeless from this event, while the destruction was estimated to cost between 10 and 15 billion USA dollars [5]. The Beirut explosion generated seismic waves equivalent to a magnitude 3.3 earthquake, according to the United States Geological Survey (USGS) Earthquake Hazard Program [6].

From the early beginning, several agencies have tried to support ground rescue investigations. Examples of this effort are by the Center for Satellite Based Crisis Information (ZKI) of the German Aerospace Centre (DLR) [7] that used high-resolution WorldView-2 multispectral images acquired just a day after the event. The Advanced Rapid Imaging and Analysis (ARIA) team at NASA’s Jet Propulsion Laboratory and California Institute of Technology in Pasadena, California, in collaboration with the Earth Observatory of Singapore (EOS), have also processed Sentinel-1 radar images indicating areas that are likely damaged caused by the explosion in Lebanon [8].

**Figure 1:**
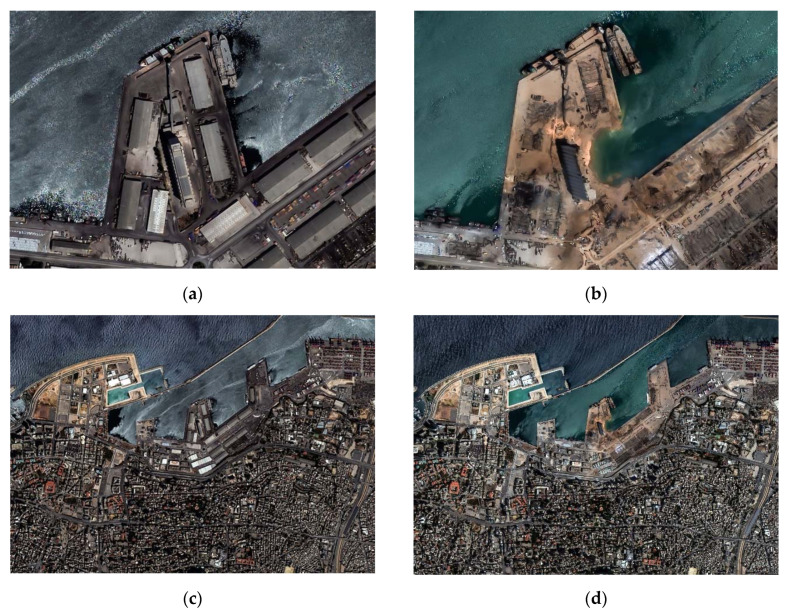
Top: (**a**) WorldView-2 high-resolution optical image over the Beirut harbor area before the explosion and (**b**) after the event. Bottom: (**c**) WorldView-2 high-resolution optical image over the broader area of the Beirut harbor before the explosion and (**d**) after the event (copyrights European Space Imaging [9]).

Figure 1 shows high-resolution red-green-blue (RGB) WorldView-2 images over the area of the harbor (Figure 1, top) and the broader area of the explosion (Figure 1, bottom) before (Figure 1a,c) and after (Figure 1b,d) the event. These images were released some hours after the explosion by the European Space Imaging [9], thus allowing visualization of the destruction.

While the above-mentioned results are very important for local stakeholders as they provide damage proxy maps in a short time, the detection of damaged areas is usually based on the processing on either optical or radar products. In this paper, we aim to investigate the Beirut explosion that took place on 4th of August 2020 by using in parallel both optical and radar Copernicus Sentinel data. The detection of damage areas in different wavelengths of the spectrum can further strengthen the final damage proxy maps.

The paper is organized as follow: initially, the methodology and the data used are presented, while the results from the change detection and InSAR analysis from the Sentinel-1 datasets are shown. These results are also compared with optical Sentinel-2 images as well as other published—in the media—high-resolution results. Finally, a general discussion for the potential of space-based applications for monitoring areas under threat is presented.

## 2. Study Area

**Figure 2:**
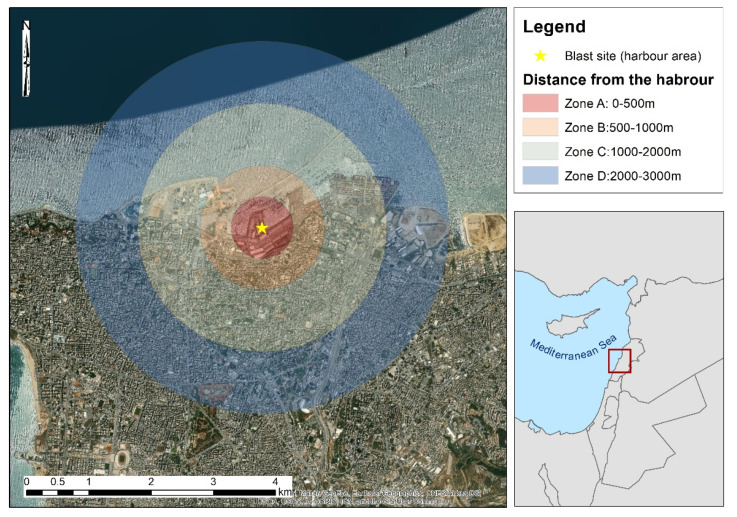
Study area indicating the harbor area (blast site) with a yellow star and the various zones created for further consideration covering distances from 0 to 3000 m away from the blast site.

The study area was focused on the harbor area of Beirut in Lebanon, where the explosion occurred, and its surrounding area (Figure 2). While much damage was observed in the harbor area (see also Figure 1a,b), other damages have been also reported and mapped from high-resolution satellite images in an area 2000 m away from the blast zone, by the DLR team (see [7]). In this study, we extended the area of interest to cover a circular area of a radius of 3000 m around the harbor area. Four main zones were defined as follows: Zone A is an area up to 500 m from the blast site, Zone B is an area from 500 m to 1000 m from the site of the explosion, and Zone C covers areas up to 2000 m away from the harbor. Finally, Zone D is defined as the area from 2000 to 3000 m away from the harbor site. The northern part of the study area is the Mediterranean Sea; therefore, no images analysis was carried out at this part.

## 3. Materials and Methods

### 3.1. Methodology

For the needs of the study, Copernicus radar freely distributed datasets were used. Once the available images were selected from the Alaska Satellite Facility (ASF) services [10], these were further processed by the Hybrid Pluggable Processing Pipeline (HyP3) cloud platform [11] (see more in Section 3.3).

The radar processing at the HyP3 cloud platform included two products as follows. A change detection map based on a pair of Radiometrically Terrain-Correct (RTC) Sentinel-1 data and an Interferometric Synthetic Aperture Radar (InSAR) analysis map, using both ascending and descending Sentinel-1 images, was used to map changes in the area. For the first product, the log difference for both VV (vertical transmit, vertical receive) and VH (vertical transmit, horizontal receive) backscattering gamma 0 amplitude polarizations of the RTC Sentinel-1 images was estimated based on the following equation for each image pixel:

Change detection = Log10 (D2/D1),(1)

where D1 refers to the Sentinel-1 with the earlier acquisition date (before the explosion) and D2 refers to the Sentinel-1 image taken after the explosion. Due to the small temporal window of the images, any changes can be linked to the blast event. A significant change of the gamma-0 amplitude can indicate areas of damage. Positive values indicate an increase in radar backscatter from the first date to the second, while negative values indicate a decrease. It should be mentioned that Sentinel-1 images are first filtered using the Enhanced Lee speckle filter, while the images are corrected based on the input Digital Elevation Model (DEM) of the area of interest. Then, the images are co-registered and radiometrically corrected (removal of radiometric distortions). The log difference of the gamma-0 amplitude for the VV and VH polarizations input images is estimated following Equation (1).

In addition, the coherence values were also mapped over the area. The degree of coherence is defined as the normalized complex correlation coefficient of the complex backscatter intensities S_1_ and S_2_ [12]. Coherence values can be estimated using Equation (2), where the * denotes the complex conjugate.

γ = |〈S_2_ × S_1_*〉/√(〈S_1_ × S_1_*〉〈S_2_ × S_2_*〉)| (2)

Coherence values may range from 0 to 1; the larger the number, the higher the coherence. A low coherence value may indicate areas that have changed between the two overpasses of the Sentinel-1 radar sensors.

The InSAR analysis of an ascending and a descending pair of Sentinel-1 images was executed—as mentioned before in the HyP3 platform—using the Gamma software. In short, the InSAR Gamma algorithm for the Sentinel-1 images comprises of eleven (11) steps as follows. (1) determine the overlapping area using the two Sentinel-1 images. (2) Download the European Union Digital Elevation Model (EU-DEM) v1.1, a hybrid Shuttle Radar Topography Mission digital elevation model (SRTM), and ASTER Global Digital Elevation Map (ASTER GDEM) data fused by a weighted averaging approach over the area of interest [13]. (3) Create a lookup table between the DEM and Sentinel-1 imagery. A lookup table for SLC co-registration, considering terrain heights, is created. The data are filtered with adaptive data filtering (ADF). (4) Create a differential interferogram using the DEM height along with the co-registration with the DEM. (5–6) Removal of the flat earth phase (5) and the topographic phase (6). (7) Refinement of the slave image (prior to the event) with the master image (after the event). A check for convergence is performed using the azimuth offset as a limit (less than 0.02 pixels). (8) Resampling of the slave to match the master image. (9) Create the final interferogram. (10) Unwrap the phase using the Minimum Cost Flow (MCF) algorithm. The MCF algorithm permits a global automatic optimization robust phase unwrapping, taking into consideration disconnected areas of high coherence. (11) Geocode the results. The end products, as well as the sub-products developed through this processing chain of the InSAR analysis, are also available for downloading by the end-user (see more in [11]).

Then, the final products from the change detection and the InSAR analysis were imported into a Geographical Information System (GIS), namely the ArcGIS v.10 software, whereas zonal statistics per each zone, namely Zone A to Zone D, were estimated. The overall results generated a damage detection proxy map that was also compared with available high-resolution RGB WorldView-2 images as well as other reports from international agencies, namely the German Aerospace Center (DLR) and the ARIA teams. In addition, Sentinel-2 optical images, taken before and after the explosion, were downloaded from the Sentinel Data Hub [14] in order to compare the results from the Sentinel-1 analysis.

### 3.2. Datasets

As mentioned earlier, two pairs of Sentinel-1 images were used for the change detection and the InSAR analysis. Table 1 indicates the characteristics of the two Sentinel-1B Interferometric Wide (IW) images used for the change detection analysis (see Equation (1)). An image with an acquisition date of the 24th of July 2020 and an image taken on the 5th of August 2020 were used. The images were taken from the same sensor (S1B) as well as with the same pass direction (ascending orbit) to minimize any noise.

In addition, two pairs of Sentinel-1B images in descending and ascending orbits were processed for the InSAR analysis. The images were taken on the 31st of July 2020 and the 6th of August 2020 with a descending orbit (see Table 2), while another pair of Sentinel-1B images in ascending orbit, with an overpass at the 29th of July 2020 and the 10th of August 2020, was used (see Table 3).

Finally, Sentinel-2 images of Level 2A (Bottom of Atmosphere (BOA) reflectance images) with a spatial resolution of 10/20 m acquired before (24th of July 2020) and after (8th of August 2020) the explosion, with limited cloud coverage over the area of interest were downloaded from the Sentinel Data Hub. The RGB optical composite of the Sentinel-2 image taken after the explosion and the NIR-R-G pseudo color composite of the same image were used for comparison purposes through interpretation with the Sentinel-1 image analysis.

In addition, Principal Component Analysis (PCA) was applied to the integrated Sentinel-2 dataset (images of 24th of July 2020 and 8th of August 2020 together) to detect any significant spectral changes near the harbor area. The PCA is a well-known statistical analysis process that takes into account the spectral variations within the image [15]. While this type of analysis is implemented in single image processing, it can also be tested for a multi-temporal dataset, whereas the temporal variance will be taken into consideration. Therefore, the PCA can be used as a fast change detection method, in cases where the radiometric noise and the time span between the images is minimum [16,17]. In our example, we used Sentinel 2 optical images of Level 2A processing, meaning that the images are geometric, radiometric, and atmospherically corrected. Therefore, the PCA can explain the changes due to the explosion over the area.

### 3.3. Big-Data Cloud Platform

The HyP3 big data cloud platform provided by the ASF was used to process the radar Sentinel-1 datasets. The platform is designed on Amazon cloud services, and upon submitting the request form, the end users may select several products and processing algorithms. Further details for the potentials of this platform can be found in [11]. The final products are distributed through Amazon’s simple storage service (S3) and are available to the end users for downloading. The HyP3 platform currently operates in a beta version, and its access is limited to restricted users. It runs a series of different radar processing chains such as Interferometric SAR (InSAR), Radiometric Terrain Correction (RTC), and change detection. The processing of the radar images is based on either the Sentinel Toolbox [18] or Gamma software [19].

## 4. Results

This section provides an overview of the results generated for this analysis of the radar images. These results are also compared with optical Sentinel-2 data as well as other high-resolution images such as those of WorldView-2 and the public products from the DLR and ARIA teams.

### 4.1. Sentinel-1 Analysis

Below, the results from the Sentinel-1 radar images is provided, following the change detection image analysis in Section 4.1.1 and the InSAR analysis in Section 4.1.2.

#### 4.1.1. Sentinel-1 Change Detection Analysis

**Figure 3:**
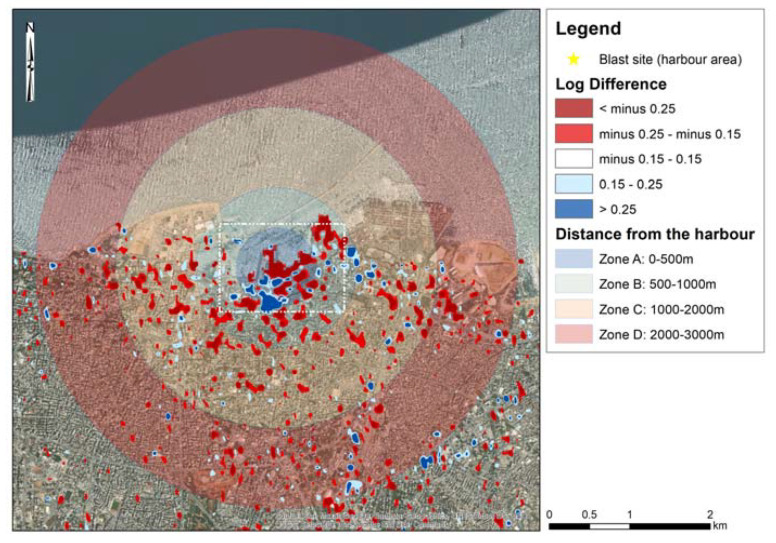
Change detection results using the log difference of the VV and the VH backscattering amplitude. Significant changes of the pair of images are presented with dark red and blue colors (>−0.25 or >0.25 differences), while other minor changes in the range of −0.25 to −0.15 and 0.15 to 0.25 are also presented in light red and blue colors, respectively. The four zones under study (Zone A to Zone D) are also given. The location of the blast size is shown with the yellow star at the center of the figure. The white dashed rectangle around the blast site is indicating the zoom area presented in Figure 4.

**Figure 4:**
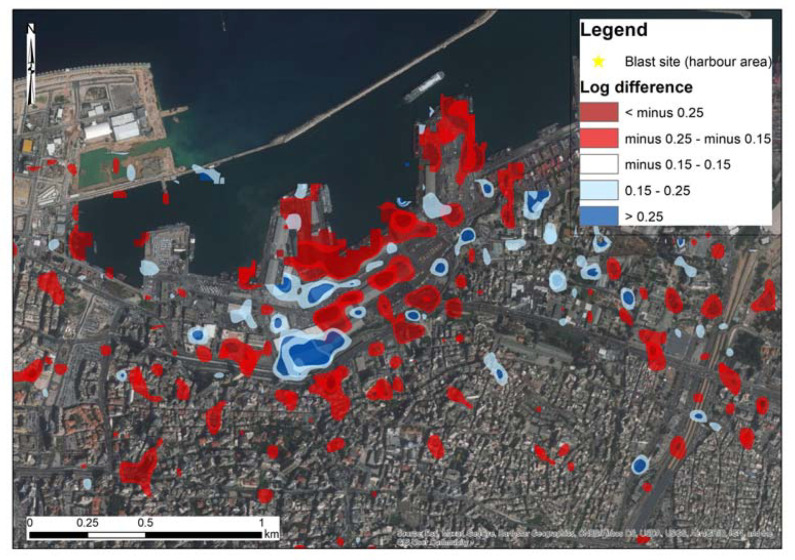
Change detection results—as in Figure 3, see before—for the area around the blast site near the harbor of Beirut (indicated with a white dashed line in Figure 3).

**Figure A1:**
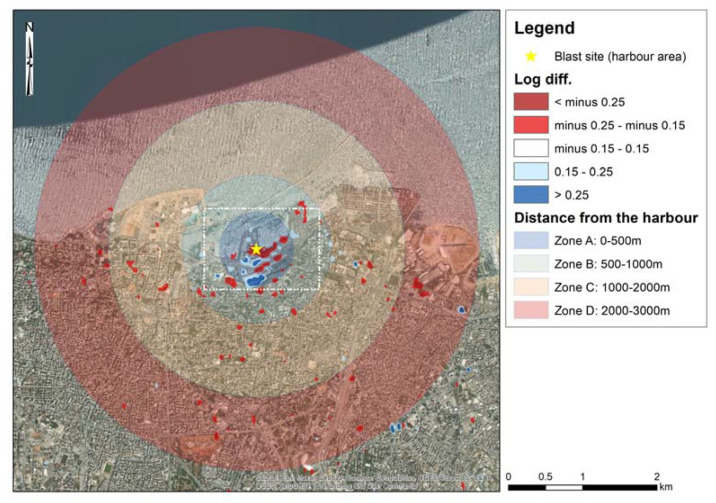
Change detection results using the log difference of the VH backscattering amplitude. Significant changes of the pair of images are presented with dark red and blue colors (>−0.25 or >0.25 differences), while other minor changes in the range of −0.25 to −0.15 and 0.15 to 0.25 are also presented in light red and blue colors, respectively. The four zones under study (Zone A to Zone D) are also given. The location of the blast size is shown with the yellow star. The white dashed rectangle around the blast site is indicating the zoomed-in area presented in Figure A3.

**Figure A2:**
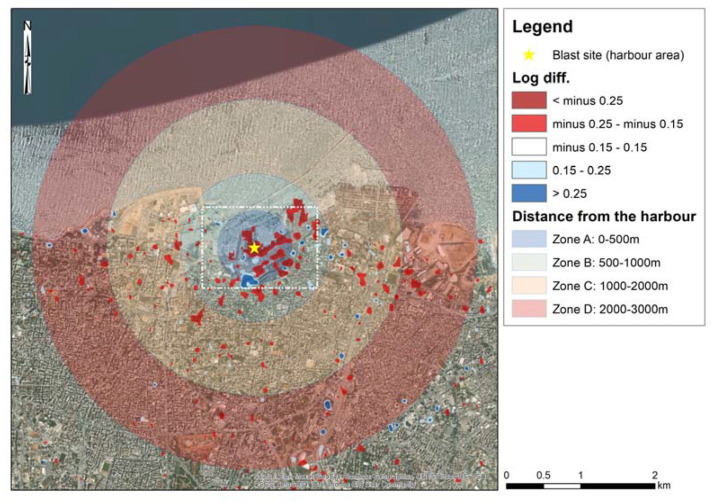
Change detection results using the log difference of the VV backscattering amplitude. Significant changes of the pair of images are presented with dark red and blue colors (>−0.25 or >0.25 differences), while other minor changes in the range of −0.25 to −0.15 and 0.15 to 0.25 are also presented in light red and blue colors, respectively. The four zones under study (Zone A to Zone D) are also given. The location of the blast size is shown with the yellow star. The white dashed rectangle around the blast site is indicating the zoomed-in area presented in Figure A4.

**Figure A3:**
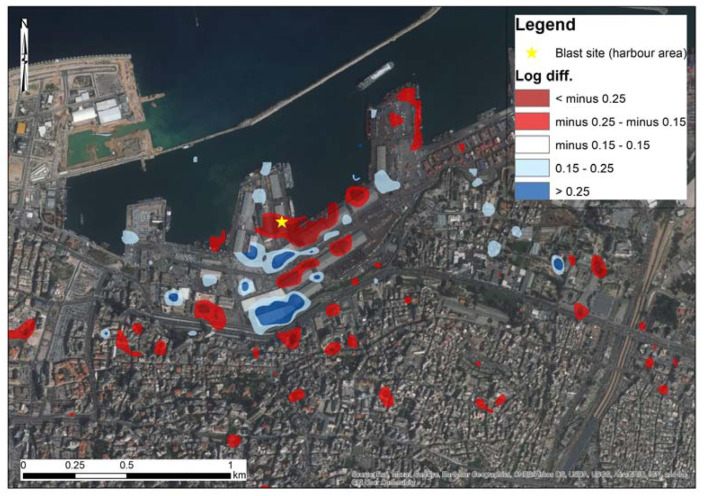
Change detection results as shown in Figure A1 (see before) for the area around the blast site near the harbor of Beirut (indicated with a white dashed line in Figure A1). The location of the blast size is shown with the yellow star.

**Figure A4:**
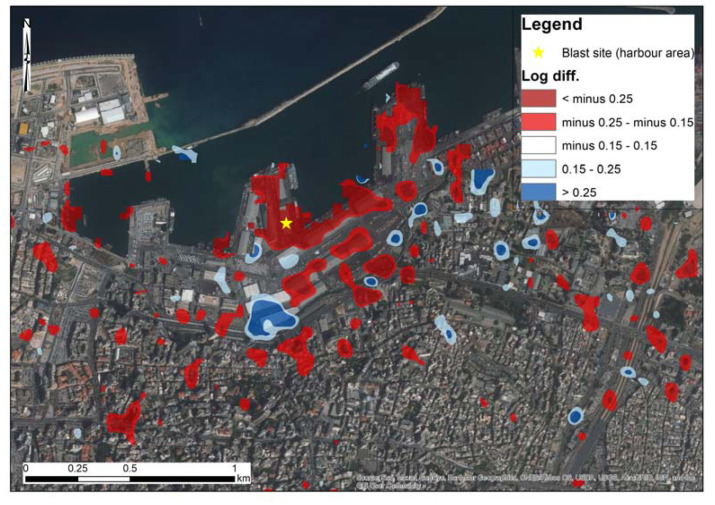
Change detection results—as in Figure A2, see before—for the area around the blast site near the harbor of Beirut (indicated with a white dashed line in Figure A2). The location of the blast size is shown with the yellow star.

Figure 3 presents the results of the change detection analysis. The two Sentinel-1B images with acquisition dates of 24th of July 2020 and 5th of August 2020 (Table 1) have been processed based on Equation (1). Two threshold values were selected to classify the change detection results for both VV and VH polarizations of the Sentinel-1 images. The default value of −0.25 and +0.25 from the HyP3 platform was kept, while another lower threshold of −0.15 and +0.15 was also applied to visualize any minor changes in the magnitude of the backscattered signals of the two images. Areas that undergo a negative change between the two images, and therefore recorded a decrease in the backscatter returns, are displayed in red, while those displaying a positive change that indicates an increase in backscatter returns are displayed in blue color. In Figure 3, the four zones under study (Zone A to Zone D) are also presented. Figure 3 is a focus of the results around the harbor area (blast site) indicating the same results. The extent of Figure 4 is indicated in Figure 3, with a white dash line around the blast site. As the individual products from the VV and the VH change, detection polarizations tend to give different accuracy levels; these can be found in Appendix A. However, although the VH polarization signals (Figure A1 and Figure A3) tend to have much lower coherence than the VV polarization signals (Figure A2 and Figure A4), both of them were able to indicate areas of changes near the blast zone.

Integrating both these polarization results, the majority of the differences of the backscattered signal (VV and VH polarizations) are around the blast zone mainly in Zone A and Zone B (Figure 3 and Figure 4). This is also evident in the focus area in Figure 4. However, damages have also been recorded on a broader area mainly to the east and south of the blast site, extended in Zones C and D, and in some areas even beyond Zone D (>3000 m). It is also important to highlight that the majority of these changes are considered quite significant (>−0.25 or >0.25, indicated with red color in Figure 3 and Figure 4), which are the result from the sudden catastrophe around the harbor zone. Details for the individual results of the VV and the VH polarizations can be found in Appendix A (see Figure A1, Figure A2, Figure A3 and Figure A4).

Table 4 summarizes the damaged areas for each zone (Zone A to Zone D) as estimated from the GIS environment using the log difference of the VH polarizations (see also Figure A1 and Figure A3). The area of destruction for each zone is estimated in hectares units (10,000 m^2^). As shown, the majority of the damaged area is found in Zone A with an area of 17.9 hectares, which equals 37.2% of the total damaged area, while another 24.5% of the total damage area is reported in Zone B that extends up to 1000 m away from the blast site. Zonal statistics analysis indicate a similar pattern for Zone C and Zone D with an approximately average damage area of 9 hectares (18.5%); however, the majority from these areas are in the medium log difference threshold units (−0.25 to −0.15 and 0.15 to 0.25). Zone A also reports the highest percentage in terms of areas of having a log difference more than >−0.25 and >0.25 (17.9 hectares or 62.0% and 11.8 hectares or 76.9%, respectively).

The next table (Table 5) shows the zonal statistics for the log difference of the VV polarizations (see also Figure A2 and Figure A4). The VV polarizations reported a larger area of damage (99.0 hectares) against the 48.2 hectares of the VH polarization analysis (Table 4). Zone A reports the highest percentage of the top threshold value (>−0.25) with an area of 14.4 hectares out of the total 25.8 hectares of Zone A. It is worth noting also that statistics from all zones (Zone A to Zone D) tend to give similar total areas of damage ranging from 20.9 hectares (Zone D) to 26.9 hectares (Zone C).

Synthesizing the results from Table 4 and Table 5, we can see the overall changes both for the VV and the VH polarizations. It should be mentioned that the new outcome that corresponds to the total damaged areas (shown in Table 6) is not the sum of Table 4 and Table 5, but the statistics after the spatial union of these tables. This affects the overlapping areas, whereas the higher-ranking threshold was kept. Based on the findings of Table 6, we can estimate the total damaged areas within 3000 m away from the blast site to be around 117 hectares. In detail, an area of 29.0 hectares was mapped in Zone A (0−500 m from the blast site), while an area of approximately 32.0 hectares was mapped for Zones B and C (500–1000 m and 1000−2000 m from the blast site, respectively). Finally, an area of 24.2 hectares was estimated in Zone D (2000−3000 m).

The majority of the damaged areas are found between a medium threshold value (from −0.25 to −0.15 and 0.15 to 0.25 log difference of the VV and VH polarization) with a total area of 84.4 hectares (57.4 and 27.0 hectares, respectively) that equals 72.1% of the total damaged area. The remaining 27.9% of the damaged area (32.5 hectares) is found at the higher threshold values (>−0.25 and >0.25).

#### 4.1.2. Sentinel-1 InSAR Image Analysis

Following the change detection analysis, the InSAR analysis for mapping the micromovements of the area because of the generated seismic waves from the explosion was performed. As mentioned earlier for the needs of the InSAR analysis, a pair of Sentinel-1B Single Look Complex (SLC) images was used, with acquisition dates of 31st July 2020 and the 6th of August 2020.

**Figure 5:**
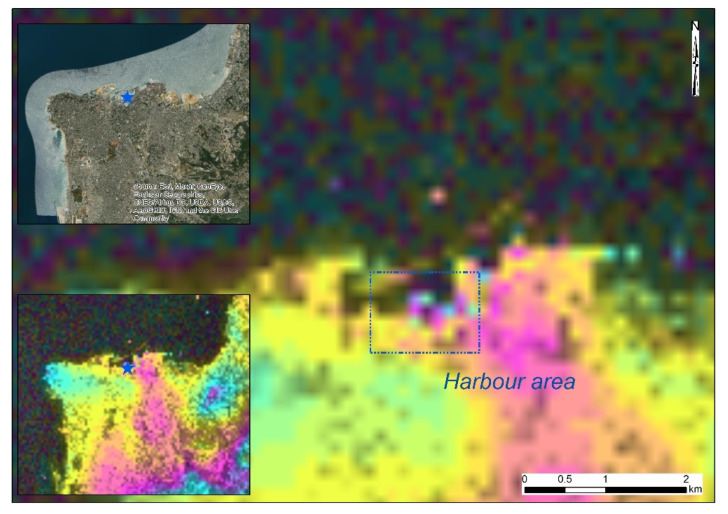
Wrapped interferogram showing the deformation fringes as a result of the Beirut explosion as derived from the Sentinel-1 SAR images. The color shows a full 2π cycle of phase change.

Figure 5 shows the wrapped interferogram around the harbor site for the descending orbit (see Table 2). The wrapped interferogram is proportional to the difference in path lengths for the SAR Sentinel-1 image pair [12]. Interferometric fringes, visible in Figure 5, represent a full 2π cycle of phase change. The deformation fringes, visible around the area of the harbor in Figure 5, can be linked to the ground movement of that area due to the explosion. It should be mentioned that this observation is only visible in the area around the harbor site. The relative ground movement between the blast site and any other point of the area can be calculated by counting the fringes and multiplying them by half of the wavelength. The closer together the fringes, the higher the deformation on the ground.

**Figure 6:**
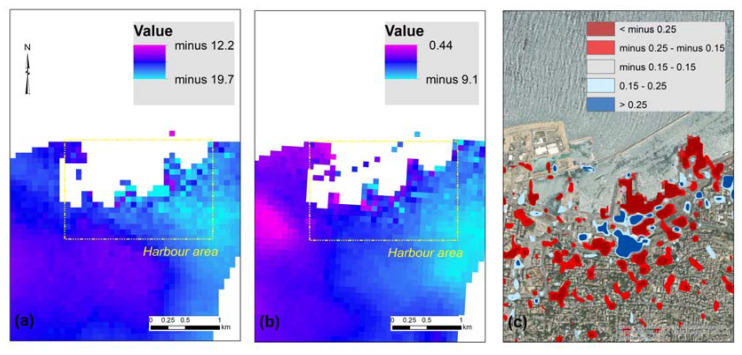
(**a**) Unwrapped interferogram as derived from the Sentinel-1 SAR images in descending orbit (see Table 2). (**b**) Unwrapped interferogram as derived from the Sentinel-1 SAR images in ascending orbit (see Table 3), and (**c**) VV and VH log difference polarizations of the same area (as in Figure 3). Harbor area is indicated in a yellow square.

Based on this wrapped interferogram, which is mainly useful for visualization purposes, the unwrapped interferogram was calculated for the descending orbit Sentinel-1 images (Figure 6a), which corresponds to the change in the distance along the line of sight of the sensor. An unwrapped interferogram converts the wrapped 2-π scale into a continuous scale (of multiples of pi). The Minimum Cost Flow (MCF) and triangulation methods (see more in [20]) are applied through the HyP3 platform for the phase unwrapping process.

Figure 6b shows the results from the ascending orbit Sentinel-1 images. Values greater than zero (positive) indicate relative movement away from the sensor (subsidence), while negative values indicate movement toward the sensor (uplift). Figure 6c shows the same area with the results from the change detection analysis. The ground relative displacements around the area of the harbor (see the yellow rectangle in Figure 6a,b) were estimated between −15 mm relative displacement along with the line of sight of the sensor for the descending orbit and −5 mm for the ascending orbit.

**Figure 7:**
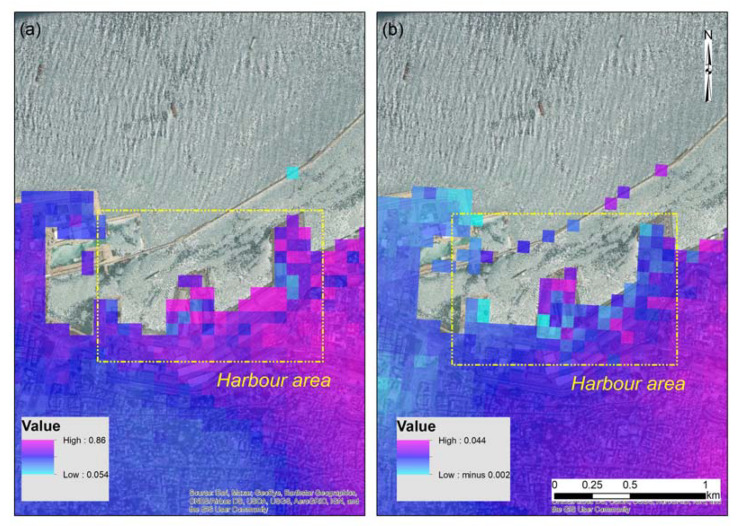
(**a**) Line of sight (LOS) displacements as derived from the Sentinel-1 SAR images (descending orbit). Harbor area, as indicated in a yellow square. (**b**) Line of sight (LOS) displacements as derived from the Sentinel-1 SAR images (ascending orbit). Harbor area, as indicated in a yellow square.

The line of sight (LOS) displacement map was estimated for both the ascending and the descending pairs of SLC Sentinel-1 (Figure 7a,b respectively). For estimating the LOS, we convert the unwrapped differential phase (Figure 6) into measurements of ground movement along the look vector (line of sight). Positive values indicate movement toward the sensor (such as uplift), while negative values indicate movement away from the sensor (such as subsidence).

In the area of interest, and based on the descending orbit Sentinel-1 images (see Table 2), we can observe only positive movements (toward the sensor) that range between 5 and 9 cm; however, the more significant movement is found in the area over the harbor area. Similar findings were also retrieved from the analysis of the ascending orbit Sentinel-1 images of Table 3. These results are shown in Figure 7b. Once again, the positive values indicate uplift, while the negative values indicate subsidence. Specific pixels within the harbor area indicate the higher relative displacements.

Of course, these examples should be taken with great caution, as a low coherence is reported at this site due to the VV and VH changes observed from the explosion, as well as the sensitivity of the Sentinel-1 sensors (in relation to their wavelength that corresponds to ≈5.54 cm). However, the findings here are quite noteworthy, as even with low coherence, the InSAR analysis was able to detect the blast site and locate significant changes, such as those reported from the change detection analysis.

**Figure 8:**
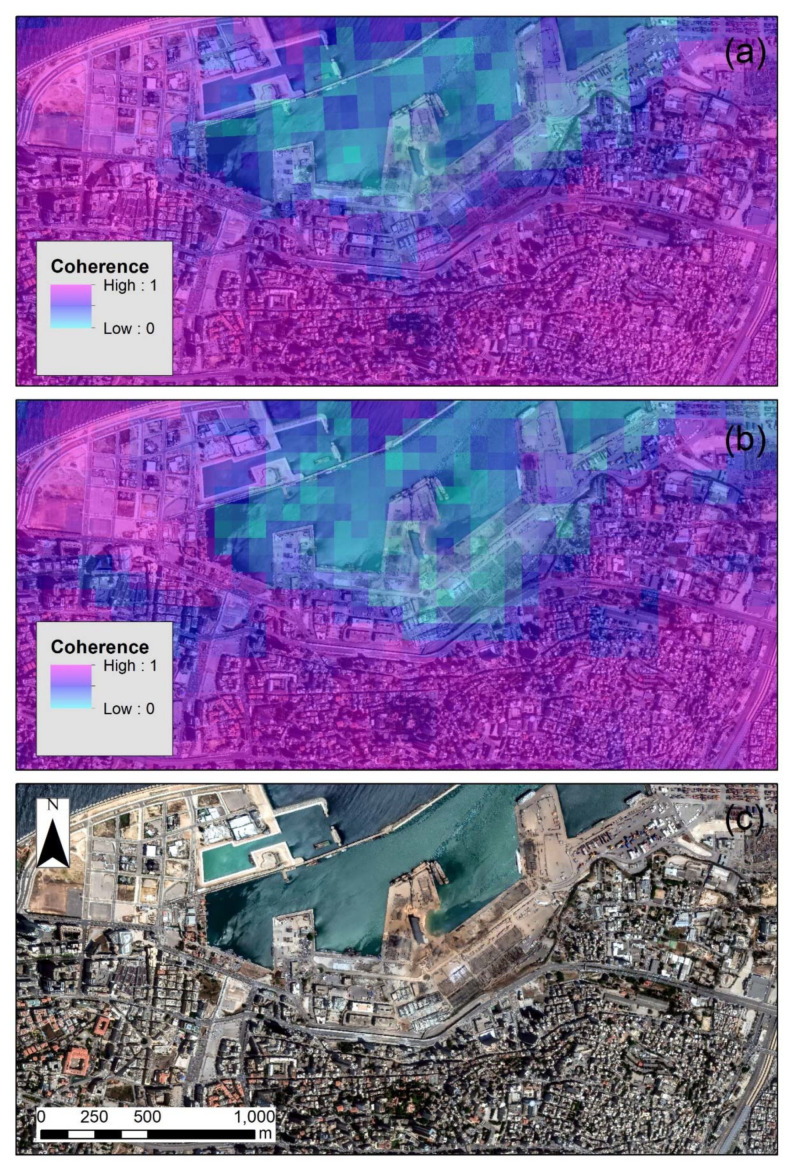
(**a**) Coherence map generated from the descending orbit images (see Table 2). (**b**) Coherence map generated from the ascending orbit images (see Table 3). (**c**) High-resolution WorldView-2 image.

The coherence map for both descending and ascending orbits are shown in Figure 8a,b, respectively. Areas highlighted with purple color indicate high coherence values, while the blue color indicates regions with low coherence. The latest (regions with low coherence), as shown in both Figure 8a,b, are around the blast zone at the Beirut harbor, indicating a significant degree of change between the two overpasses of the Sentinel-1 (both in the ascending as well as for the descending orbits).

### 4.2. Sentinel-2 Image Analysis

**Figure 9:**
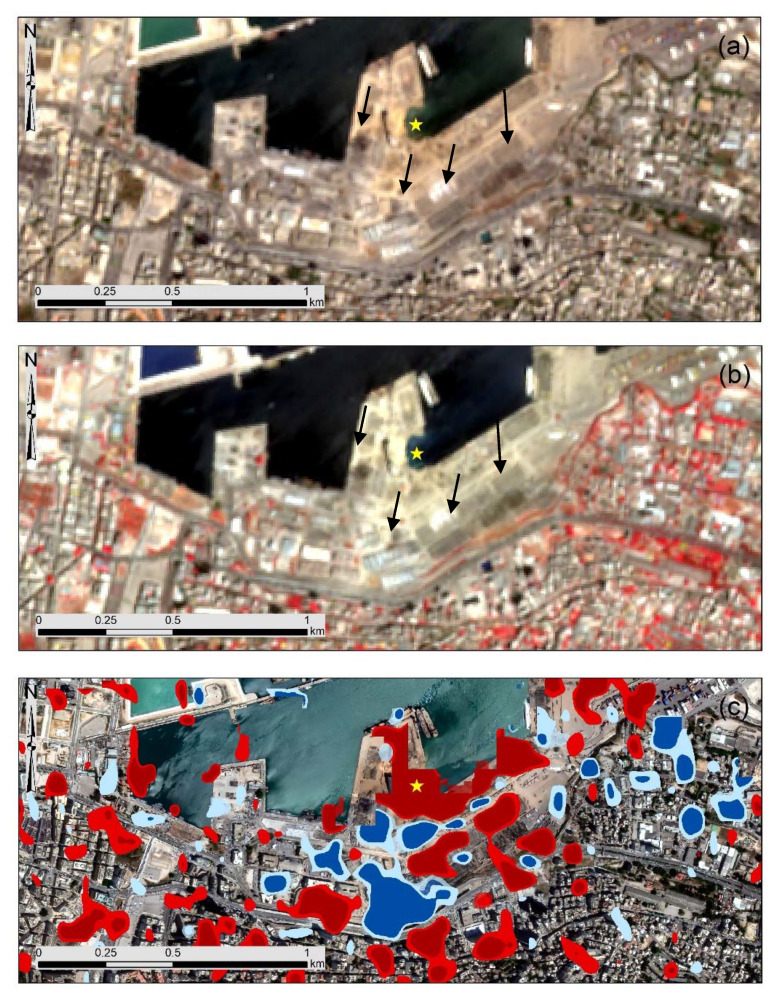
(**a**) Sentinel-2 optical image taken over the area of interest on the 8th of August 2020 (RGB composite). (**b**) The NIR-R-G pseudo color composite of the same image and (**c**) the change detection results from the Sentinel-1 image analysis. Black arrows indicate destroyed areas from image interpretation of the Sentinel-2 image, while yellow arrows indicate the non-detectable destroyed areas from this analysis. The location of the blast size is shown with the yellow star.

The results from the Sentinel-1 images have also been compared with the Sentinel-2 optical image taken after the 4th of August 2020. The RGB composite of the image is shown in Figure 9a. Destroyed buildings can be observed in the area near the exposition site; however, these are more evident in the NIR-R-G pseudo color composite shown in Figure 9b. Vegetated areas are shown with red color in this figure. The correlation between the visual inspection of the Sentinel-2 and the Sentinel-1 change detection results can be seen in Figure 9c.

Visual interpretation of the optical image can depict some of the most apparent damages over the area (see black arrows in Figure 9).

**Figure 10:**
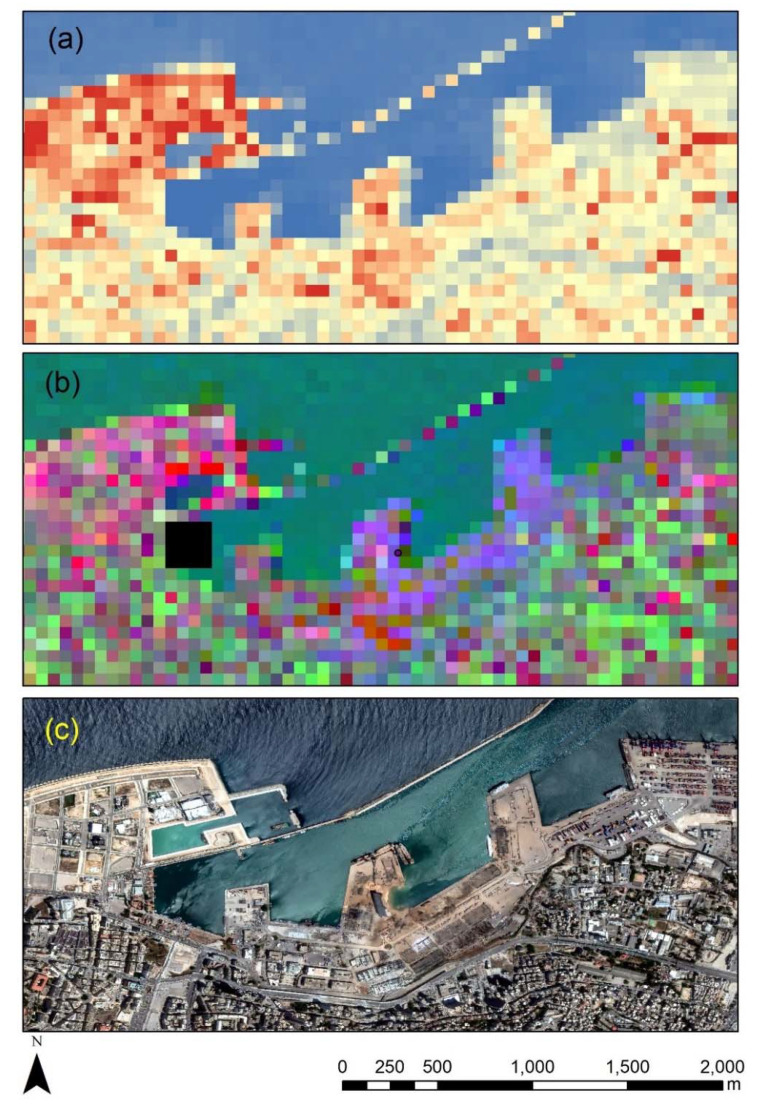
(**a**) The first principal component (PC1) results over the area around the harbor after the Principal Component Analysis (PCA) of the integrated Sentinel-2 optical images of 24th of July 2020 and 8th of August. (**b**) The PC1–PC3 pseudo color composite of the same integrated dataset and (**c**) the high-resolution WorldView-2 image.

To further process the optical Sentinel-2 images, PCA was implemented in the integrated datasets of the Sentinel-2 images taken on 24th of July 2020 and 8th of August 2020. The results are shown in Figure 10. Figure 10a shows the results from the first principal component (PC1) while Figure 10b shows an RGB pseudo color composite of PC1–PC3. Figure 10c shows the high-resolution optical WorldView-2 image over the area. The red color in Figure 10 shows higher PC1 values, thus indicating changes in the integrated temporal Sentinel-2 dataset (i.e., 24th of July 2020 and 8th of August 2020 together). As evident, higher PC1 values are located around the blast area as well as in the western part of the harbor. Similar findings can be also reported from the pseudo color composite where the first three principal components, namely PC1 to PC3, have been used.

## 5. Comparison with Other Results

The explosion at Beirut harbor has left significant and extensive damage in the surrounding buildings but also in other areas far away from the blast site. In the previous section, we have presented how this destruction could be detected by the Copernicus Sentinel-1 sensor via two difference analyses: (a) a change detection approach and (b) an InSAR analysis. The overall results were also confirmed through other studies that have been reported only some hours after the event, such as the case of the ARIA and the DLR teams. In addition, the availability of a high-resolution WorldView-2 image taken over the area of interest has confirmed the results generated from the analysis of the Sentinel-1 sensors.

**Figure 11:**
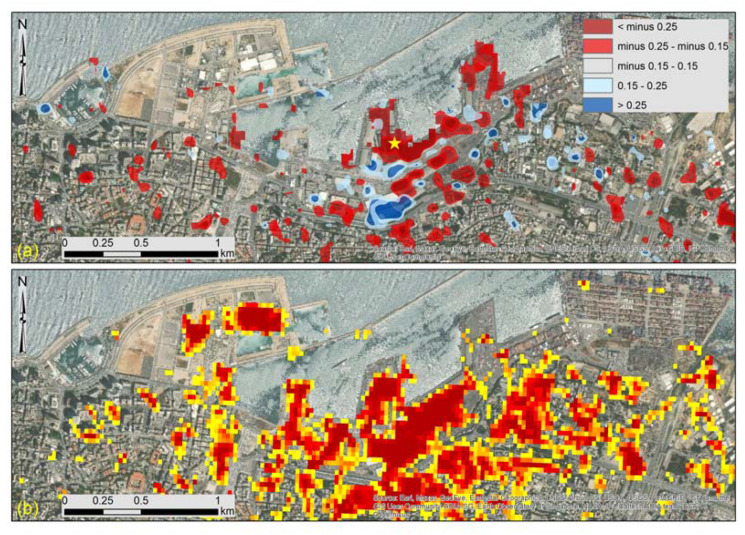
(**a**) Change detection results as generated from the VV and VH log differences of the Sentinel-1 images, while (**b**) indicates the Damage Proxy Map generated from the Advanced Rapid Imaging and Analysis (ARIA) team [8]. The location of the blast size is shown with the yellow star.

To evaluate the overall performance of this study, we have compared the results presented above with other published material (maps) and available high-resolution WorldView-2 images. Initially, we have compared our change detection results (Damage Proxy Map) with the one of the ARIA team [8], as these have been generated with the Sentinel-1 sensor (as here). The comparison is shown in Figure 11. Figure 11a shows the change detection results with the threshold used in our study, while Figure 11b shows the damaged area as reported from the ARIA team. Although there is no threshold unit for this classification, it was evident that a similar pattern is reported from both studies, while adjusting the thresholds values would generate similar results. Once again, the area with the most damages is the one around the blast site.

**Figure 12:**
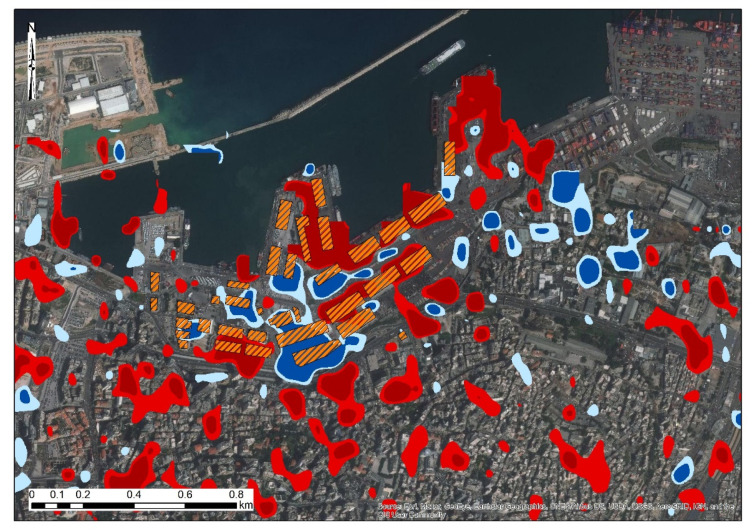
Change detection results as generated from the VV and VH log differences of the Sentinel-1 images around the blast site, while orange polygons indicate the buildings that have been damaged as reported by the MapAction platform [21] (digitized by the author).

Within the area around the harbor of Beirut, digitization of the buildings that have been damaged has been reported by the MapAction platform [21]. Similar findings with this report are also found in the maps generated by the DLR team, which are not shown here. Figure 12 shows the focus area, whereas the VV and the VH log difference polarizations (as found from this study) are presented, and the digitized results from the MapAction platform. Comparing these two results, we can see a high correspondence between the two products; however, these were generated from different resolutions.

**Figure 13:**
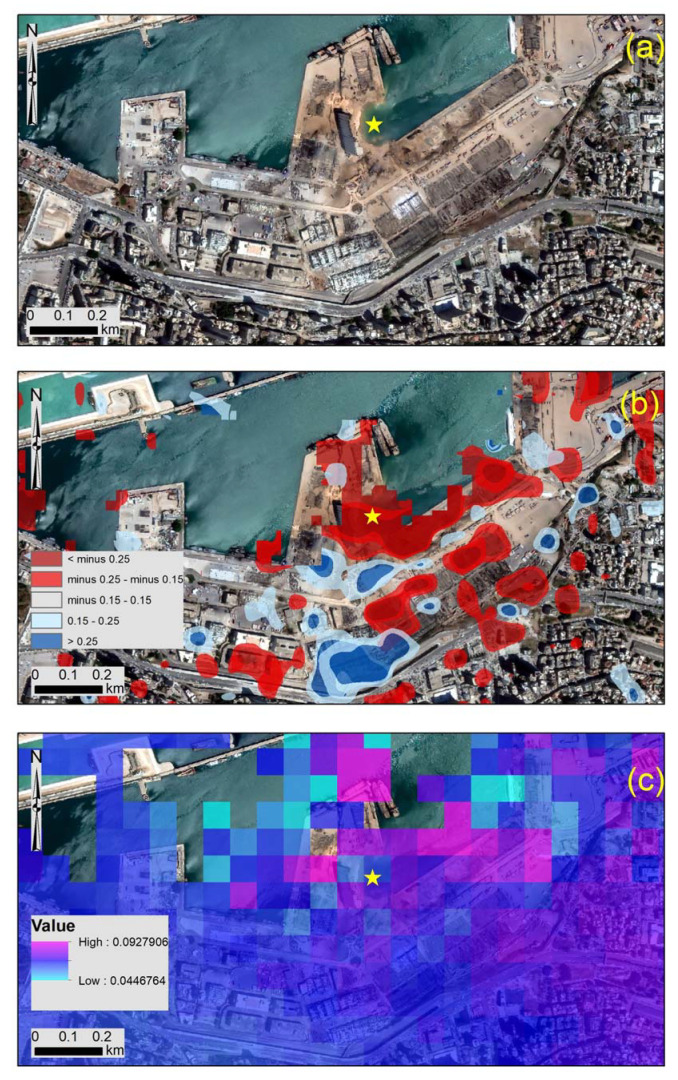
(**a**) High-resolution WorldView-2 image taken some hours after the exposition over the harbor of Beirut. The blast site is indicated with a yellow star. (**b**) Change detection as generated from the VV and VH log differences of the Sentinel-1 image and (**c**) the InSAR analysis (LOS of the descending orbit images) over the area. The location of the blast size is shown with the yellow star.

A direct comparison of the high-resolution WorldView-2 image that was taken just after the explosion over the harbor of Beirut was carried out. This image is shown in Figure 13a, whereas the destroyed buildings can be observed in the image. The blast site, indicated with a yellow star in Figure 13a, has now been vanished due to the highly explosive chemicals that were kept in the site. Both the change detection analysis (Figure 13b), as well as the InSAR analysis (Figure 13c), shows a good correlation with the visual inspection of the high-resolution WorldView-2 image despite the difference in their spatial resolution (0.30 m of the WorldView-2 image against the 10m resolution of the Sentinel-1 results).

## 6. Conclusions

A damage proxy map based on the processing of Sentinel-1 images was carried out in this study over the harbor area of Beirut, Lebanon after the high explosion occurred in the area on the 4th of August 2020. The analysis of this study was achieved through a change detection analysis of Sentinel-1 images, while the seismic waves results were also mapped using an InSAR Sentinel-1 image analysis.

The analysis was able to be carried out due to the systematic observations of the Copernicus Sentinel-1 sensors operated by the European Space Agency (ESA), while the processing chain was carried out by the big-data cloud HyP3 platform operated by ASF. This combination allows end users to process in a short time (less than 1-h computational time) a series of radar processing chains in almost global coverage. Of course, this is feasible due to the free and fully open policy (FFO) of the Sentinel datasets.

The findings of this study are well aligned with other products delivered by specialized space centers such as the DLR and the ARIA teams, while a comparison was also performed using a high-resolution WorldView-2 image. While the medium-resolution Sentinel-1 images and their products do not allow us to detect individual destruction in a building level, the 10-m resolution is enough for estimating with high accuracy the damaged area. The use of Sentinel-2 images could support the detection analysis through visual interpretation of significant changes in the landscape as well as through a PCA temporal analysis. This was more obvious using the NIR-R-G pseudo color composite; however, due to the spatial resolution of the image (20 m), the detection of smaller areas was quite challenging.

The use of both sensors, namely the Sentinel-1 and Sentinel-2, can be further utilized in the future to support emergency situations, with an almost global coverage. In this case, other events not so well known can be monitored by the scientific local community to support specific emergency needs. Of course, these maps can be only indicative of the destruction, as ground verifications are needed. In addition, the analysis of the results from this series of analyses should be seen with great caution, as the images might suffer from other factors that influence the final outcomes. For instance, the relative micromovements can be due to other sources, such as the atmospheric component and even DEM accuracy, while the change detection of the VV and the VH polarizations need to be linked also with their coherence.

Overall, the use of Sentinel data was found very promising for supporting ground investigations after an event (such as the one presented here, or even a natural hazard). Future steps can include considering the automation of the whole procedure, minimizing the time between the data processing and the event itself.

## Figures and Tables

**Table 1:** Sentinel-1 data used for the change detection analysis.

Name	Characteristics Prior to the Event (Reference-D1)	Characteristics After the Event (Secondary–D2)
Date	The 24th of July 2020	The 5th of August 2020
Mode	Interferometric Wide swath (IW)	Interferometric Wide swath (IW)
Satellite	Sentinel-1B	Sentinel-1B
Absolute Orbit number	22,615	22,790
Pass direction	ASCENDING	ASCENDING
Polarization	VV + VH	VV + VH
Product type	ground range (GRD)	ground range (GRD)
Path	14	14
Frame	107	107

**Table 2:** Sentinel-1 data used for the Interferometric Synthetic Aperture Radar (InSAR) analysis (descending orbit).

Name	Characteristics Prior to the Event (Reference—D1)	Characteristics After the Event (Secondary—D2)
Date	The 31st of July 2020	The 6th of August 2020
Mode	Interferometric Wide swath (IW)	Interferometric Wide swath (IW)
Satellite	Sentinel-1B	Sentinel-1B
Absolute Orbit number	33,693	22,797
Pass direction	DESCENDING	DESCENDING
Polarization	VV + VH	VV + VH
Product type	Single Look Complex (SLC)	Single Look Complex (SLC)
Path	21	21
Frame	480	480

**Table 3:** Sentinel-1 data used for the InSAR analysis (ascending orbit).

Name	Characteristics Prior to the Event (Reference—D1)	Characteristics After the Event (Secondary—D2)
Date	The 29th of July 2020	The 10th of August 2020
Mode	Interferometric Wide swath (IW)	Interferometric Wide swath (IW)
Satellite	Sentinel-1B	Sentinel-1B
Absolute Orbit number	22,688	22,863
Pass direction	ASCENDING	ASCENDING
Polarization	VV + VH	VV + VH
Product type	Single Look Complex (SLC)	Single Look Complex (SLC)
Path	87	87
Frame	107	107

**Table 4:** Zonal statistics using the VH polarization for each log difference threshold and zone (Zone A to Zone D). Areas are measured in hectares (10,000 m^2^).

Log Difference	Zone A	Zone B	Zone C	Zone D	Total
>−0.25	4.4	0.5	0.6	1.6	7.1
−0.25 to −0.15	4.4	7.2	6.2	5.6	23.4
0.15 to 0.25	5.5	3.8	1.8	1.9	13.0
>0.25	3.6	0.4	0.3	0.5	4.7
Total	17.9	11.8	8.9	9.5	48.2

**Table 5:** Zonal statistics using the VV polarization for each log difference threshold and zone (Zone A to Zone D). Areas are measured in hectares (10,000 m^2^).

Log Difference	Zone A	Zone B	Zone C	Zone D	Total
>−0.25	14.4	6.0	3.8	2.5	26.7
−0.25 to −0.15	6.0	12.6	16.1	10.7	45.5
0.15 to 0.25	3.1	4.7	5.3	5.9	19.0
>0.25	2.3	2.1	1.7	1.7	7.8
Total	25.8	25.4	26.9	20.9	99.0

**Table 6:** Zonal statistics using the synthesis of the VH and VV polarizations for each log difference threshold and zone (Zone A to Zone D). Areas are measured in hectares (10,000 m^2^).

Log Difference	Zone A	Zone B	Zone C	Zone D	Total
>−0.25	11.0	5.2	4.1	2.9	23.3
−0.25 to −0.15	8.3	17.0	19.6	12.6	57.4
0.15 to 0.25	5.9	7.8	6.4	6.9	27.0
>0.25	3.7	2.1	1.6	1.9	9.2
Total	29.0	32.0	31.7	24.2	116.9
